# The Epidemiology of Premenstrual Syndrome and Sports Injuries Among National-Level Adolescent Female Badminton Athletes

**DOI:** 10.3390/healthcare14060778

**Published:** 2026-03-19

**Authors:** Zhuo Chen, Kazuhiro Imai, Baiyang Ding, Xiao Zhou, Eiji Watanabe, Katsuji Aizawa

**Affiliations:** 1 National Academy of Education Administration, Ministry of Education, Beijing 102617, China; 2 School of Public Administration, Sichuan University, Chengdu 610065, China; 3 Department of Life Sciences, Graduate School of Arts and Sciences, The University of Tokyo, Tokyo 153-8902, Japan; dingboyang_yi12@yeah.net; 4 School of Physical Education, Huazhong University of Science and Technology, Wuhan 430074, China; 5 Institute of Sport, Senshu University, Kawasaki 214-8580, Japank.aizawa@isc.senshu-u.ac.jp (K.A.)

**Keywords:** premenstrual symptoms, female players, knee injury, lumbar injury

## Abstract

**Background**: Physical activity is widely acknowledged as an effective intervention for alleviating premenstrual syndrome (PMS) symptoms. However, the optimal exercise dosage that provides symptom relief without increasing the risk of sports-related injuries remains unclear, particularly in adolescent females. **Objectives**: This study investigates the epidemiology of PMS and sports injuries among national-level adolescent female badminton athletes to inform training practices that balance symptom relief and injury prevention. **Methods**: A total of 63 national-level adolescent female badminton players aged 13–18 completed questionnaires assessing medical history related to badminton, premenstrual symptoms (PSQ), and menstrual health. All participants were adolescent females who had experienced menarche. Data were analyzed to identify associations between training parameters, PMS symptoms, and injury prevalence. **Results**: The analysis showed that 13.8% of female badminton athletes experienced knee and lumbar injury associated with badminton participation. The peak incidence of knee injuries occurred at the age of 15, whereas the age of 17 was a peak period for lumbar injuries. A total of 47 (74.6%) athletes experienced at least one premenstrual symptom. The most common symptoms were anger/irritability and anxiety/tension. The players with premenstrual syndrome showed significantly increased menses time (OR: 1.16, *p* < 0.05), menstrual days in the past 12 months (OR: 4.06, *p* < 0.05), and badminton experience period compared with those without (OR: 1.441, *p* < 0.05). **Conclusions**: PMS symptoms and sports-related injuries are prevalent among national-level adolescent female badminton athletes. Longer participation history in badminton was associated with a higher prevalence of PMS symptoms. These findings underscore the importance of considering menstrual health alongside injury prevention in the training and health management of adolescent female athletes. Further studies using objective training exposure and clinical assessments are warranted.

## 1. Introduction

Many women experience physical and mood changes in the days leading up to menstruation. When these symptoms persist month after month and interfere with daily life, they are referred to as premenstrual syndrome (PMS) [1]. The prevalence of PMS varies across different regions: a study found that 21.1% of Chinese adult women experienced PMS [2], while the prevalence among Japanese adult women was only 5.3% [3]. Additionally, the incidence rates for PMS severity are as follows: mild PMS affects 11.3–93.5% of women, moderate PMS affects 4.6–5.3%, and severe PMS affects 1.2–2.1% [3].

Premenstrual syndrome (PMS) affects a substantial proportion of women and can significantly disrupt daily life. Approximately 20% of adult women report serious impairment due to PMS symptoms [2]. Interestingly, the incidence of moderate to severe PMS is reportedly higher among high school students compared to adult women [3]. These symptoms may also affect adolescents’ tolerance to physical effort and training stress, particularly during the premenstrual phase [4]. However, the characteristics of PMS in adolescent female athletes remain poorly understood. Physical activity is widely recognized as an effective intervention for alleviating PMS symptoms in adolescent females [5]. However, high-intensity training loads are associated with physiological stress, changes in energy utilization efficiency, and endocrine system fluctuations, which might contribute to menstrual irregularities and symptom exacerbation [6]. Therefore, examining the relationship between training-related factors and PMS is useful for gaining insights into health management within populations of adolescent female athletes. In addition, evidence addressing the interaction between growth-related factors, training participation, and PMS across different adolescent age stages remains limited.

Badminton is a popular sport played worldwide. According to the Nippon Badminton Association’s 2019 member data, Japan has 303,743 registered badminton players, of which, 203,907 (63.7%) are junior high or high school students [7]. An epidemiological badminton study reported that 49.6% to 82.0% of the youth badminton players experienced at least one badminton injury [8,9,10]. Elevated injury rates were observed in the lumbar spine, the dominant knee joint, and the dominant shoulder joint [11]. The incidence of injuries rate per 1000 h ranges from 0.9 to 5.1 in badminton players [12]. The injury rate during practice was higher in women than men and increased with age [11]. Previous studies also reported that increased age, female sex, increased badminton hours weekly, and low badminton skill level were risk factors for badminton injury [8,13,14]. Higher injury rates during the luteal phase and the first few days of the menses were reported possibly due to impaired postural balance, coordination and proprioception [15,16]. Another study demonstrated significantly greater postural sway that decreased with PMS symptoms [17].

Despite the growing body of literature on PMS and sports-related injuries, evidence focusing specifically on adolescent female badminton athletes remains limited. In particular, epidemiological data describing the prevalence of PMS symptoms and badminton-related injuries, as well as their associations with badminton participation characteristics, are scarce in this population. Moreover, the extent to which prolonged engagement in competitive badminton is associated with PMS symptoms and injury occurrence during adolescence has not been well documented. Therefore, the aim of this study was to investigate the epidemiology of PMS symptoms and sports-related injuries and to explore their associations with badminton participation history among national-level adolescent female badminton athletes aged 13–18 years.

## 2. Materials and Methods

### 2.1. Participants

The participants in this study were junior school-aged badminton players (aged 13–18 years) belonging to the Japan Schoolchildren Badminton Federation and participating in the national tournament. This study was reviewed and approved by the Ethical Committee of the Graduated School of Arts and Sciences, University of Tokyo, Japan (Notification Number 602–2 July 26, 2018). The study protocol was performed in compliance with the tenets of the Declaration of Helsinki.

A self-reported, paper-based questionnaire was independently completed by 75 national-level female junior badminton players after guardian consent was obtained in December 2022. Three paper questionnaires were used to collect data, consisting of medical information related to badminton, the menstrual situation of the female badminton players, and their premenstrual symptoms (PSQ). Among all the participants, we excluded 12 individuals who had not yet experienced menarche. Finally, 63 participants completed all three questionnaires.

### 2.2. Data Collection

The first questionnaire, designed to assess the medical status of the badminton players, comprised two sections. The first section collected information on the players’ physical characteristics—such as age, sex, weight, and height—and their training history, including the number of years involved in badminton, daily training hours, and number of training days in a week. The second section addressed the players’ lifetime injury and pain history across 25 anatomical regions, such as the head, knee, and lumbar area. The Female Athlete Menstrual Situation Questionnaire includes questions about the age at menarche, the use of analgesics, the frequency of menstruation per year, the duration of each menstrual period, and self-assessment of symptoms at various menstrual stages. The Premenstrual Symptoms Questionnaire (PSQ) translates DSM-IV (Diagnostic and Statistical Manual of Mental Disorders, Fourth Edition) criteria into a severity rating scale in Japanese. For this study, PMS was diagnosed based on the criteria established by the American College of Obstetricians and Gynecologists (ACOG).

The PSQ inquired whether, within the past three months, participants experienced the following premenstrual symptoms starting a week before menstruation and subsiding a few days after its onset. The questionnaire includes PMS symptoms categorized into affective and somatic types. The affective symptoms are: depression, anxiety or tension, difficulty concentrating, tearfulness, anger or irritability, and decreased interest in work or social activities. The somatic symptoms are: fatigue or lack of energy, overeating or food cravings, insomnia or hypersomnia, feeling overwhelmed, and physical symptoms such as tender breasts, bloating, headaches, joint or muscle pain, and weight gain. Additionally, the PSQ assesses whether these PMS symptoms affected work efficiency, home responsibilities, social activities, or relationships with coworkers or family. Symptoms are rated as not at all, mild, moderate, or severe.

### 2.3. Statistical Analysis

All statistical analyses were conducted using SPSS for Windows, Version 26.0 (IBM Corp., Armonk, NY, USA). Descriptive statistics were used to summarize the demographic and training characteristics of the participants, as well as the prevalence of PMS symptoms and injury rates. Normality testing of continuous variables (e.g., age, height, weight, BMI) was performed using the Shapiro–Wilk test. Normally distributed data are presented as means ± standard deviations, while non-normally distributed data were expressed as medians and interquartile ranges (IQRs). Comparative analyses: Student’s t-test was used to compare continuous variables between participants with and without PMS symptoms. Binary logistic regression was used to examine the association between training-related variables (e.g., training duration and frequency) and the presence of PMS. Multivariate logistic regression was conducted to adjust for potential confounders (e.g., age, BMI, and years of training) when analyzing the relationship between PMS and injury occurrence. The significance level was set at *p* < 0.05 and 95% confidence intervals (CIs) were calculated for all odds ratios to estimate the precision of the associations.

## 3. Results

**Figure 1:**
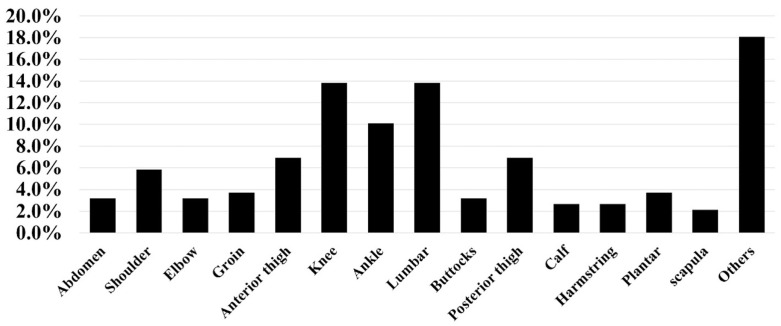
The distribution of injuries in all female badminton athletes.

**Figure 2:**
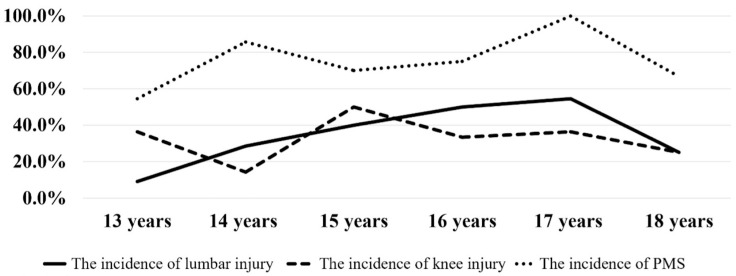
The incidence of lumbar injury, knee injury, and PMS among Japanese female badminton athletes in different age groups.

A total of 63 national-level female adolescent badminton players met the criteria. The most commonly injured areas were the knees and lumbar region (Figure 1). The age of 15 was the peak age for the incidence of knee injuries, with a prevalence rate of 50.0% among individuals in this age category. The peak age for the incidence of lumbar injury was identified as 17 years, with a prevalence rate of 54.4% within this age group (Figure 2).

According to the results of PSQ, 47 (74.6%) out of 63 of the national-level female adolescent badminton players experienced PMS symptoms and were grouped into a PMS-related symptoms group, and the players without PMS symptoms were grouped into a No-PMS-related symptoms group. The prevalence of each PMS symptom among all the participants is shown in Table 1. The most common mood symptom was anger/irritability and anxiety/tension, with 65.1% of them having experienced both of these symptoms. The most common physical symptom was fatigue or lack of energy, with 58.7% of them having experienced this symptom. Meanwhile, the most severe psychological symptoms and physical symptoms were anger or irritability and overeating or food cravings, respectively (11.1%). Premenstrual symptoms impaired “work efficiency or productivity, home responsibilities” in 34.9% of the participants, “social life activities” in 15.9%, and the relationship with coworkers or family in 11.1% (Table 2).

The baseline characteristics are shown in Table 3. The association between each variable and the PMS-related symptoms was analyzed using logistic regression. The PMS group showed significantly increased menses times in the past 12 months compared with the No-PMS group. In the PMS group, menses occurred 9.94 times/year and were 1.16 times more likely compared to the No-PMS group (9.94 vs. 6.53, OR: 1.16, 95% CI: 1.023–1.305, *p* < 0.05). Regarding menstrual days in the past 12 months, participants with less than 25 menstrual days were 4.06 times more likely to experience PMS than those with more than 36 menstrual days (OR: 4.06, 95% CI: 1.16–14.26, *p* < 0.05). The PMS group showed more badminton experience (9.46 years vs. 7.58 years, OR: 1.441, 95% CI: 1.073–1.934, *p* < 0.05).

## 4. Discussion

The results of this study found that the prevalence of PMS-related symptoms was 74.6%. This proportion exceeds that reported for normal Japanese students and athletes measured using the same PSQ scale [4]. Specifically, the incidence of PMS among female adolescents was reported to be 57.3%. Variations in PMS incidence across different studies may be attributed to the use of different diagnostic scales, as well as the inclusion of diverse age ranges and sociocultural and socioeconomic backgrounds [18,19,20]. Additionally, younger women are reported to have a higher risk of PMS, likely due to the condition’s increasing prevalence with age and its close association with ovulatory cycles. In adolescents, establishing regular ovulatory cycles may require maturation of the hypothalamic–pituitary–ovarian (HPO) axis over time following menarche [21]. This might help explain the higher incidence of PMS-related symptoms observed among adolescent females in our study compared to their general peers. Furthermore, excessive exercise might be a risk factor for various injuries and can lead to uncontrollable physical and psychological symptoms [22,23]. Although exercise is often promoted as a universal remedy for various health issues, it is crucial to evaluate the evidence critically. The tendency to recommend exercise as a solution for menstrual disorders such as PMS and primary dysmenorrhea lacks substantial support and requires careful scrutiny and balanced endorsement [24]. Training load comprises three elements: training intensity, training duration and frequency, and duration of sports participation. This study investigated self-reported training duration and frequency, as well as duration of sports participation. Direct training intensity was not measured and was not available. Future research should investigate exercise intensity using methods such as heart rate or the session Rating of Perceived Exertion (RPE) scale.

The female athletes’ triad consists of osteoporosis, disordered eating and menstrual disorders, which are detrimental to the performance and health of athletes [25]. Reproductive irregularities in female athletes frequently stem from disruptions in hypothalamic function and the pulsatile release of gonadotropin-releasing hormone (GnRH) [26]. Although the advantages of regular exercise on health are well-documented, the impact of excessive physical activity on psychological and physical health has not been fully understood, and the guidelines for the optimal amount of exercise have not yet been defined [27]. Regarding the relationship between exercise volume and health, determining the adequate amount of exercise is key. It is important to avoid both excessive exercise and insufficient exercise. A previous study measured the dose–response relationship between children aged 4–18 years, and found that lower levels of physical activity were associated with diminished academic achievement and cognitive performance when contrasted with their physically active counterparts [28,29,30,31,32,33]. We previously reported that for students aged 13–17, lack of exercise might be a risk factor for PMS [34]. It is also possible that menstrual irregularity is common during adolescence due to ongoing maturation of the hypothalamic–pituitary–ovarian (HPO) axis, and associations with PMS symptoms in this age group may therefore reflect physiological variability rather than pathology [35]. A significant correlation existed between engaging in physical activity more than twice a week and a lower incidence of PMS. The associations observed in this study between training-related factors and PMS symptoms might reflect the physiological and psychological stress associated with training. Because this study employed a cross-sectional design, it is impossible to demonstrate a direct causal relationship between training-related factors and PMS symptoms, which is a limitation. However, the findings provide preliminary evidence suggesting that training-related characteristics might be associated with the severity of PMS symptoms in adolescent female athletes. This study suggests that higher levels of exercise exposure are not necessarily associated with additional health benefits, highlighting the potential importance of exercise quantity in relation to health. More studies are needed to reveal the appropriate exercise time, intensity, and frequency.

The principal finding of our study is a knee injury incidence rate of 13.8%, which is consistent with the general youth population but on the lower end of the 10–35% range that was previously reported [36]. This variation in outcomes could be due to the limited sample size in our study, the characteristics of badminton, or other factors that have yet to be explored, providing directions for further research. In this study, the incidence of PMS and injuries differed depending on age. Specifically, PMS and lumbar injuries peaked at 17 years old, while knee injuries peaked at 15 years old. This annual variation in incidence rates for PMS and injuries among female athletes aged 13 to 18 contrasts with previous findings that identified the peak age for knee injuries as 13 years [37]. One potential explanation for these discrepancies is that, at around the age of 12, neuromuscular adaptation and coordination might not keep up with the rate of skeletal growth [38]. Epidemiological studies have reported that OSD affects between approximately 7% and 30% of athletic adolescents and is closely linked with growth and development rather than isolated trauma [39]. These physiological mismatches could contribute to the higher susceptibility to injuries observed in early adolescence. Previous studies have also highlighted that female athletes are more at risk for injuries than their male counterparts. The highest injury incidence rate was observed during early adolescence, a period associated with the pubertal growth spurt, which is a vulnerable phase for both mental and physical development [4,40]. It should be noted that the injuries recorded in the present study were based on self-reported previous badminton-related injury history rather than standardized time-loss or injury severity classifications. Therefore, the observed association reflects injury occurrence in a broad sense and does not distinguish between pain-related symptoms and clinically diagnosed tissue injuries. Therefore, our findings underscore the need for greater attention to adolescent female athletes, focusing on injury prevention and tailored training programs during this critical developmental phase.

Participation in sports enhances strength, endurance, and flexibility, contributing to better weight control and an increase in self-esteem [41]. However, these benefits are offset by the potential for injuries associated with sports activities [42,43]. Our data indicated a trend toward more injuries in older grades in adolescent females. However, the reasons behind the peak in lumbar injuries at age 17 warrant further investigation. A thorough understanding of epidemiology and risk factors, and implementation of control and prevention measures are crucial for enhancing women’s health. Regarding the association section between PMS and injuries, according the relevant literature, our findings are consistent with emerging research on the multifactorial mechanisms linking PMS to elevated injury risk. Neuromuscular instability [44], hormone-induced connective tissue changes [45], and PMS-related emotional dysregulation all contribute to a plausible theoretical basis [46]. These mechanisms underscore the importance of considering menstrual cycle phases in athlete monitoring and injury prevention strategies.

This study offers two key contributions. First, to the best of our knowledge, there is limited research describing the epidemiology of PMS symptoms and sports injuries among national-level adolescent female badminton athletes, and this study contributes new empirical evidence in this area. Second, it provides a comprehensive epidemiological survey analyzing the characteristics of PMS and injuries in athletes, thereby enhancing our understanding of the interplay between injuries, PMS, and overall health. However, this study has several limitations. First, PMS symptoms were assessed using a retrospective self-report questionnaire rather than prospective daily symptom ratings, which may introduce recall bias and potentially overestimate the prevalence of PMS. Second, while the study identifies certain risk factors, it does not establish a causal relationship between physical activity frequency and the incidence of PMS and injuries. Third, the relatively small sample size might limit the generalizability of the findings. No a priori sample size or power calculation was performed due to the inherently limited number of national-level adolescent badminton athletes; therefore, the results should be interpreted with caution. Another limitation is that the observed associations might be influenced by unmeasured factors such as psychological stress, sleep, nutritional status, and academic workload. Therefore, these results should be interpreted with caution, and future longitudinal studies using comprehensive training load measurements are necessary to confirm these associations.

## 5. Conclusions

This study shows that sports-related injuries are relatively common among national-level adolescent female badminton athletes, with knee and lumbar injuries being the most frequently reported injuries. A total of 13.8% of the athletes experienced knee or lumbar injuries associated with badminton participation. The occurrence of these injuries exhibited clear age-specific patterns, with knee injuries peaking at the age of 15 years and lumbar injuries reaching their highest incidence at 17 years. These findings indicate that different musculoskeletal regions may be vulnerable at different stages of adolescence. Recognizing age-related injury patterns may contribute to more precise injury surveillance and monitoring in competitive youth badminton players.

Premenstrual symptoms were highly prevalent in this cohort, with 74.6% of the athletes reporting at least one symptom. Emotional symptoms, particularly anger/irritability and anxiety/tension, were the most commonly reported, suggesting that psychological manifestations constitute a major component of PMS in adolescent female badminton players. The high occurrence of PMS symptoms highlights that menstrual-related symptoms are a widespread health issue in this population rather than isolated individual cases. These findings emphasize the necessity of incorporating menstrual health indicators into routine health assessments of adolescent female athletes.

Athletes with premenstrual syndrome demonstrated significantly different menstrual and training-related characteristics compared with those without PMS. Specifically, PMS was associated with a longer menses duration, a greater number of menstrual days in the past 12 months, and a longer history of badminton participation. These associations suggest that both menstrual characteristics and cumulative sports participation may be related to the presence of PMS symptoms. Taken together, the results underscore the importance of considering menstrual health alongside sports participation history in the health management of adolescent female athletes. Further studies incorporating objective training exposure measures and clinical assessments are warranted to clarify these associations.

## Figures and Tables

**Table 1:** Prevalence and the severity of premenstrual symptoms. *n* = 63.

	Not at All	Mild	Moderate	Severe
Depressed mood, *n* (%)	37 (58.73)	19 (30.15)	5 (7.94)	2 (317)
Anxiety or tension, *n* (%)	22 (34.92)	17 (27.69)	19 (30.16)	5 (7.94)
Tearful, *n* (%)	37 (58.73)	19 (30.16)	3 (4.76)	4 (6.35)
Anger or irritability, *n* (%)	22 (34.92)	17 (26.98)	17 (27.98)	7 (11.11)
Decreased interest in work, home or social activities, *n* (%)	44 (69.84)	11 (17.46)	8 (12.70)	0 (0)
Difficulty concentrating, *n* (%)	34 (54.00)	22 (34.92)	7 (11.11)	0 (0)
Fatigue or lack of energy, *n* (%)	26 (41.27)	23 (36.50)	10 (15.87)	4 (6.35)
Overeating or food cravings, *n* (%)	27 (42.86)	16 (25.40)	13 (20.63)	7 (11.11)
Insomnia or hypersomnia, *n* (%)	37 (58.73)	15 (23.81)	9 (14.26)	2 (3.17)
Feeling overwhelmed, *n* (%)	50 (79.37)	9 (14.29)	4 (6.35)	0 (0)
Physical symptoms, *n* (%)	33 (52.38)	15 (23.81)	11 (17.46)	4 (6.35)

**Table 2:** Prevalence and severity of premenstrual symptoms affecting work, social life and family (*n* = 63).

	Not at All	Mild	Moderate	Severe
Work efficiency or productivity, home responsibilities, *n* (%)	41 (65.08)	12 (19.01)	9 (14.29)	1 (1.59)
Social life activities, *n* (%)	53 (84.13)	9 (14.29)	1 (1.59)	0 (0)
Relationships with coworkers or family, *n* (%)	56 (88.90)	6 (9.52)	1 (1.59)	0 (0)

**Table 3:** Logistic regression analysis of variables influencing exercise association with PMS symptoms.

Variable	No PMS Symptoms (16)	PMS Symptoms (47)	OR (95% CI)	*p*-Value
Age, yrs	15.58 ±1.40	16.4 ±1.78	1.34 (0.94–1.89)	0.103
Height, m	1.56 ± 0.06	1.59 ± 0.05	1.06 (0.70–1.62)	0.057
Weight, kg	50.09 ± 6.71	52.75 ± 5.78	1.08 (0.98–1.19)	0.135
BMI, kg/m^2^	20.61 ± 2.22	20.96 ± 2.01	1.09 (0.82–1.43)	0.562
Age of menarche, mean ± SD	12.50 ± 0.82	12.47 ± 1.10	0.97 (0.56–1.69)	0.914
Menses occurrences in the past 12 months, mean ± SD	6.53 ± 4.91	9.94 ± 4.70	1.16 (1.02–1.32)	0.020 *
Menstrual days in the past 12 months, *n* (%)				
<25 d	1 (6.25)	5 (10.64)	4.06 (1.16–14.23)	0.029 *
25–35 d	7 (43.75)	30 (63.83)	5.33 (0.52–54.33)	0.158
>36 d	8 (50.00)	12 (25.53)	1	
Taking painkillers, *n* (%)				
Yes	3 (18.75)	18 (38.29)	2.69 (0.67–10.76)	0.162
No	13 (81.25)	29 (61.70)		
Experience, yrs	7.58 ± 1.43	9.46 ± 2.64	1.44 (1.07–1.93)	0.015 *
Training Sessions/Week, *n* (%)				
≤5	2 (12.50)	6 (12.77)	0.98 (0.18–5.41)	0.978
>5	14 (87.50)	41 (87.23)	1	
Hours/Training Session, *n* (%)				
2	3 (18.75)	3 (6.38)	0.30 (0.05–1.90)	0.379
3	7 (43.75)	24 (51.06)	1.03 (0.30–3.56)	0.200
4	6 (37.50)	20 (42.55)	1	
Hours/Week, *n* (%)				
2	5 (31.25)	18 (38.29)	1.06 (0.26–4.32)	0.936
3	6 (37.50)	12 (25.53)	0.59 (0.15–2.38)	0.457
4	5 (31.25)	17 (36.17)	1	
Warm-up, min	28.75 ± 10.72	26.77 ± 15.59	0.99 (0.99–1.03)	0.641
Cool-down, min	16.56 ± 17.77	14.30 ± 14.67	0.99 (0.96–1.03)	0.611

Notes: Data are presented as numbers (percentages) or means ± SDs. PMS: premenstrual syndrome; yrs: years; BMI: body mass index; OR: odds ratio; CI: confidence interval; *: statistically significant difference.

## Data Availability

The datasets presented in this article are not readily available because they contain sensitive and personally identifiable information of minors. Requests to access the datasets should be directed to chenzhuo11111@gmail.com.
